# Antipredator behaviors in urban settings: Ecological experimentation powered by citizen science

**DOI:** 10.1002/ece3.9269

**Published:** 2022-09-09

**Authors:** Benjamin Zuckerberg, Jennifer D. McCabe, Neil A. Gilbert

**Affiliations:** ^1^ Department of Forest and Wildlife Ecology University of Wisconsin‐Madison Madison Wisconsin USA; ^2^ The Peregrine Fund Boise Idaho USA

**Keywords:** accipiter, citizen science, community science, experimental ecology, urban ecology, wintering birds

## Abstract

1. Animal behaviors are often modified in urban settings due to changes in species assemblages and interactions. The ability of prey to respond to a predator is a critical behavior, but urban populations may experience altered predation pressure, food supplementation, and other human‐mediated disturbances that modify their responsiveness to predation risk and promote habituation.
2. Citizen‐science programs generally focus on the collection and analysis of observational data (e.g., bird checklists), but there has been increasing interest in the engagement of citizen scientists for ecological experimentation.
3. Our goal was to implement a behavioral experiment in which citizen scientists recorded antipredator behaviors in wild birds occupying urban areas. In North America, increasing populations of *Accipiter* hawks have colonized suburban and urban areas and regularly prey upon birds that frequent backyard bird feeders. This scenario, of an increasingly common avian predator hunting birds near human dwellings, offers a unique opportunity to characterize antipredator behaviors within urban passerines.
4. For two winters, we engaged citizen scientists in Chicago, IL, USA to deploy a playback experiment and record antipredator behaviors in backyard birds. If backyard birds maintained their antipredator behaviors, we hypothesized that birds would decrease foraging behaviors and increase vigilance in response to a predator cue (hawk playback) but that these responses would be mediated by flock size, presence of sentinel species, body size, tree cover, and amount of surrounding urban area.
5. Using a randomized control–treatment design, citizen scientists at 15 sites recorded behaviors from 3891 individual birds representing 22 species. Birds were more vigilant and foraged less during the playback of a hawk call, and these responses were strongest for individuals within larger flocks and weakest in larger‐bodied birds. We did not find effects of sentinel species, tree cover, or urbanization.
6. By deploying a behavioral experiment, we found that backyard birds inhabiting urban landscapes largely maintained antipredator behaviors of increased vigilance and decreased foraging in response to predator cues. Experimentation in citizen science poses challenges (e.g., observation bias, sample size limitations, and reduced complexity in protocol design), but unlike programs focused solely on observational data, experimentation allows researchers to disentangle the complex factors underlying animal behavior and species interactions.

## Graphical abstract

We demonstrate the promise of citizen science in conducting ecological experimentation in human‐modified landscapes. Conducting a unique playback experiment, citizen scientists showed that urban birds maintained anti‐predator behaviors.

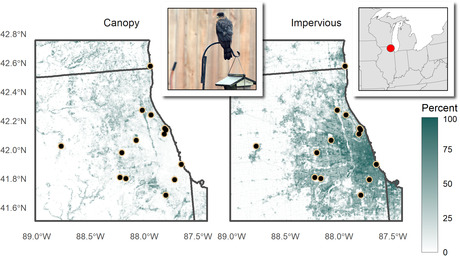

## 1 INTRODUCTION

Urban areas present several dimensions of novelty relevant to species interactions (Guiden et al., 2019). Humans alter natural habitats by building infrastructure and otherwise driving land cover changes, often resulting in less vegetative cover and simpler foliar structure (Mitchell et al., 2016; Moll et al., 2019). Additionally, human subsidies (e.g., bird feeding) result in altered congregations of animals, with implications for individual resource use, species interactions, and community assembly (Becker & Hall, 2014; Galbraith et al., 2015; Manlick & Pauli, 2020; Newsome et al., 2015; Oro et al., 2013). Predators are considered especially vulnerable to human disturbance (Estes et al., 2011), but several predator species are rebounding from historic population declines and colonizing urban areas (McCabe et al., 2018), and as a result, there is a unique opportunity to study predator–prey interactions in human‐modified landscapes (Carthey & Blumstein, 2018; Uchida & Blumstein, 2021).

In birds, antipredator behavior represents a suite of flexible behaviors for evading predators and often varies across species (Brown et al., 1999; Gaynor et al., 2019; Lima & Dill, 1990). For example, a bird may increase vigilance in response to predation risk by spending more time with its head elevated while feeding or spending less time handling food (Lima & Dill, 1990). Certain traits are often associated with interspecific variation in antipredator behaviors; for example, large‐bodied birds are generally less tolerant to human presence and will initiate flight earlier in response perceived threats (Blumstein, 2014). Some species also form flocks, which reduces individual risk due to collective vigilance, predator confusion, or risk dilution (Beauchamp, 2003; Pulliam, 1973; Roberts, 1996), resulting in decreased vigilance for individual birds within larger flocks (“group size effect”; Beauchamp, 2003). Finally, birds may “eavesdrop” on certain nearby heterospecifics—dubbed “sentinels”—that are particularly effective at detecting predators (e.g., Black‐capped Chickadees [*Poecile atricapillus*] initiate mobbing calls in the presence of a predator that other species respond to; Lilly et al., 2019; Templeton & Greene, 2007). Importantly, these antipredator behaviors are flexible, often being expressed differently in safe versus risky environments (e.g., open habitats; Griesser & Nystrand, 2009; Ware et al., 2015) and in response to changes in cost–benefit trade‐offs (e.g., favoring food acquisition in resource‐poor environments or seasons Lima, 1987; Lima & Dill, 1990). Any reduction in perceived predation risk may lead to a subsequent reduction in antipredator behavior for prey and an increase in habituation if the predator is considered absent, inefficient, or repeated encounters result in nonlethal exposures (Cooper & Wilson, 2007; Shettleworth, 2009).

In urban areas, predation pressure is often reduced due to a more diverse and abundant prey base and the presence of supplemental food sources (Fischer et al., 2012). For birds, there is mounting evidence that a decline in predation risk by native predators can produce reduced antipredator responses to predator cues. For example, flight initiation distance is an often‐used measure of sensitivity to disturbance and predation risk, and several studies have shown that birds show shorter flight initiation distances (i.e., increased tolerance to predation risk) in urban compared with rural areas (Díaz et al., 2013; Møller, 2008; Møller et al., 2013, 2015). Similarly, in a meta‐analysis of birds, mammals and lizards, Samia et al. (2015) found that birds occupying urban areas were more tolerant of disturbance than their suburban or rural counterparts. Consequently, species occupying urban areas are thought to be bolder, more habituated to disturbance and less responsive to predation risk.

**FIGURE 1:**
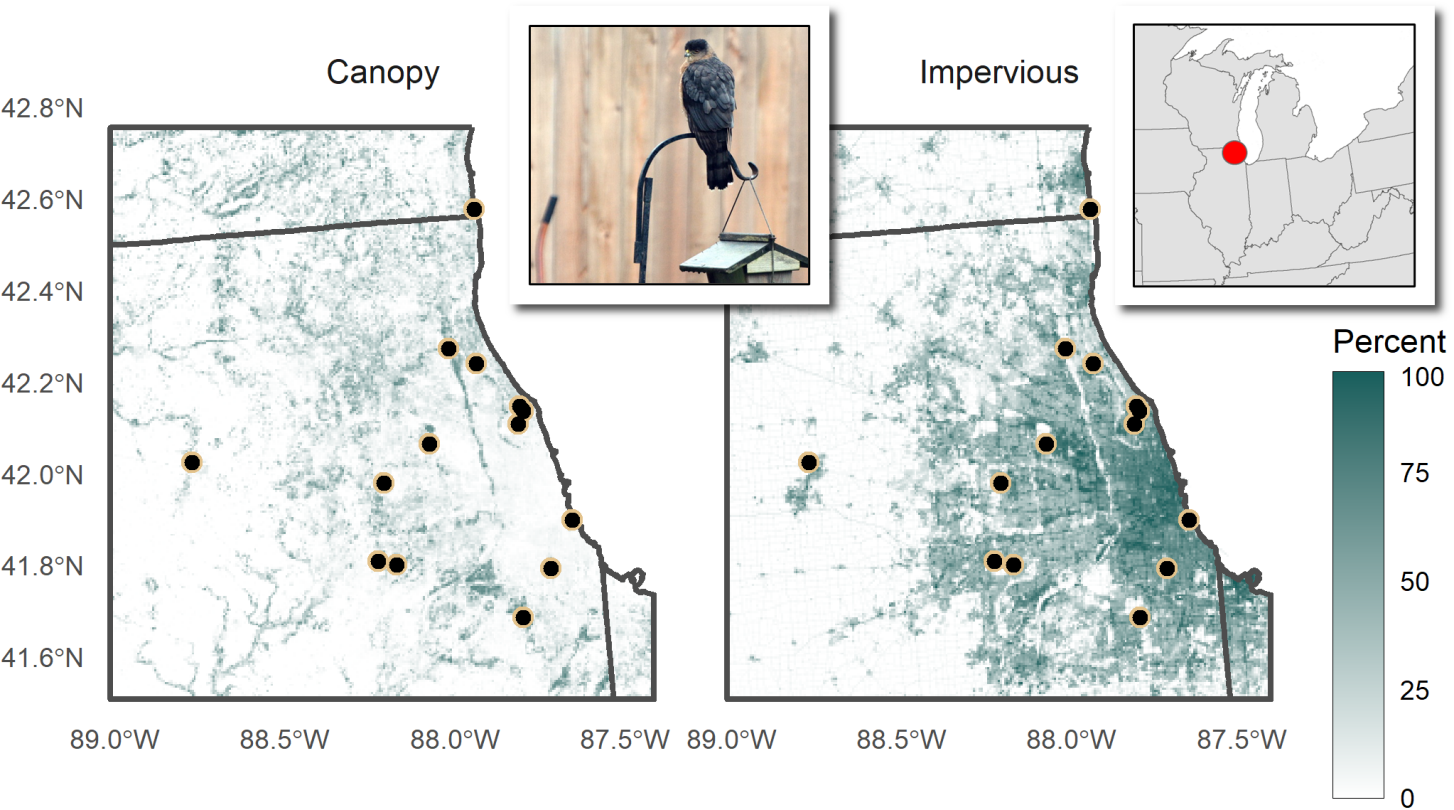
Map depicting the fifteen Project FeederWatch sites where citizen scientists collected behavioral data in Chicago, IL. Percent tree canopy cover is displayed on left (darker colors indicates a higher percentage of tree canopy cover) and percent impervious surface cover is shown on right (darker colors indicates a higher percentage). Hawk photo by Jim Culp, www.flickr.com/photos/jimculp/49380148093.

In recent years, many predators, once rare or extirpated from urban areas, are beginning to colonize and persist in urban landscapes. Across North America, sharp‐shinned (*A. striatus*) and Cooper's hawks (*A. cooperii*) were once considered sensitive to human disturbance, forest loss, and urbanization (Rosenfield, 2018), but over the last half century, hawk populations recovered and began colonizing urban areas (Rosenfield et al., 2020). The modern colonization of hawks in urban areas is presumably a response to high concentrations of their preferred prey (e.g., American robins [*Turdus migratorius*] and European starlings [*Sturnus vulgaris*]) in cities (Boal & Dykstra, 2018; Estes & Mannan, 2003; McCabe et al., 2018; Rosenfield et al., 2020). Once established in urban landscapes, hawks can reach higher densities, produce larger clutches, and switch to a more specialized diet of larger prey birds than in rural habitats (Estes & Mannan, 2003; Rosenfield et al., 1995). Over two decades, hawks colonizing urbanized areas in Chicago were able to persist in areas even with low tree cover as long as those areas supported high abundances of backyard birds (McCabe et al., 2018). Accipiter hawks typically rely on perch‐and‐scan methods to find prey (Roth II & Lima, 2003), and as such, urban backyards offer prime hunting grounds (Figure 1a). For urban‐dwelling birds, the dual effect of increased tolerance to disturbance and the increasing prevalence of natural predators offers a unique setting for exploring the consistency of predator–prey interactions.

Predator–prey interactions are complex, and thus experimentation (rather than observation) is crucial to understand antipredator behavior (Fraser et al., 2013; Smith et al., 2020). Citizen science—the involvement of the public in the collection and analysis of data related to the natural world—has revolutionized ecological research in recent decades (Cooper, 2016; Dickinson et al., 2010). While citizen science has greatly expanded the scale of scientific data collection, citizen scientists typically collect observational data. For example, many citizen‐science programs (e.g., eBird) rely on observers to record their observations of organisms in a given location and time (e.g., Sullivan et al., 2009); while these data can reveal important ecological insights, inferring causation from such observational data is challenging. An emerging opportunity in citizen science is experiments in which volunteers deploy treatments and record corresponding outcomes (Gracanin et al., 2020; Kaartinen et al., 2013). For example, Kaartinen et al. (2013) enlisted hundreds of citizen scientists and dozens of cattle farms across Finland to set up exclusion experiments to study rates of decomposition of cattle dung in pastures and found that the largest‐bodied taxon of beetles accounted for a majority (61%) of invertebrate‐caused dung decomposition. Such efforts could allow researchers to move beyond occurrence records and use data collected by volunteers to address complex biological phenomena such as species interactions and how those interactions change in human‐modified landscapes (Acuto & Parnell, 2016; Gao & O'Neill, 2020; Seto et al., 2010).

Here, we demonstrate the potential for citizen‐science experiments to elucidate complex predator–prey interactions in urban backyards. Our goal was to develop a novel citizen‐science experiment to evaluate whether songbirds retain antipredator behaviors in urban settings. We hypothesized that body mass, the presence of sentinel species, surrounding impervious surface cover (a measure of urbanization), tree canopy cover, and flock size mediate an individual's antipredator behavior. Specifically, if urban‐dwelling birds maintain their antipredator defenses, we predicted that, when exposed to a predatory cue, individual birds would be less vigilant (less responsive) in more urban landscapes, in larger flocks, closer to vegetative cover, and when a sentinel species was present. Moreover, we expected that larger species would be more vigilant to predation risk as they have higher sensitivity to potential threats (Blumstein, 2014) and are the preferred prey of *Accipiter* hawks. We evaluated these hypotheses via an experiment in which volunteers broadcast calls of Cooper's hawks and recorded the behavioral responses of their backyard birds. We conducted our study in Chicago, Illinois, USA, a major metropolitan area that has experienced recolonization by *Accipiter* hawks in recent decades (McCabe et al., 2018).

## 2 MATERIALS AND METHODS

### 2.1 Site selection: Project FeederWatch

We studied the antipredator behavior of feeder birds at 15 locations in the greater Chicago, Illinois area for two winter seasons (2016–2017 and 2017–2018; Figure 1). We solicited participants from volunteers enrolled in Project FeederWatch, a citizen science program operated by the Cornell Lab of Ornithology and Bird Studies Canada (www.feederwatch.org). The program is designed to study changes in the distribution and abundance of birds in winter across North America. Briefly, program participants record the maximum number of each species they see at their feeding stations (hereafter sites) during a 2‐day count from early November to late April (Wells et al., 1998). At the start of the two focal seasons, we emailed a project description to all FeederWatch participants living within 100 km of Chicago's city center. We chose Chicago because of its relatively high FeederWatch participation rate and its recent colonization of Cooper's and Sharp‐shinned Hawks (McCabe et al., 2018). Of the 55 and 137 participants emailed each year, 6 and 10 participated in the experiment, respectively (with one participant participating in both years).

### 2.2 Stimuli and experimental procedure

**FIGURE 2:**
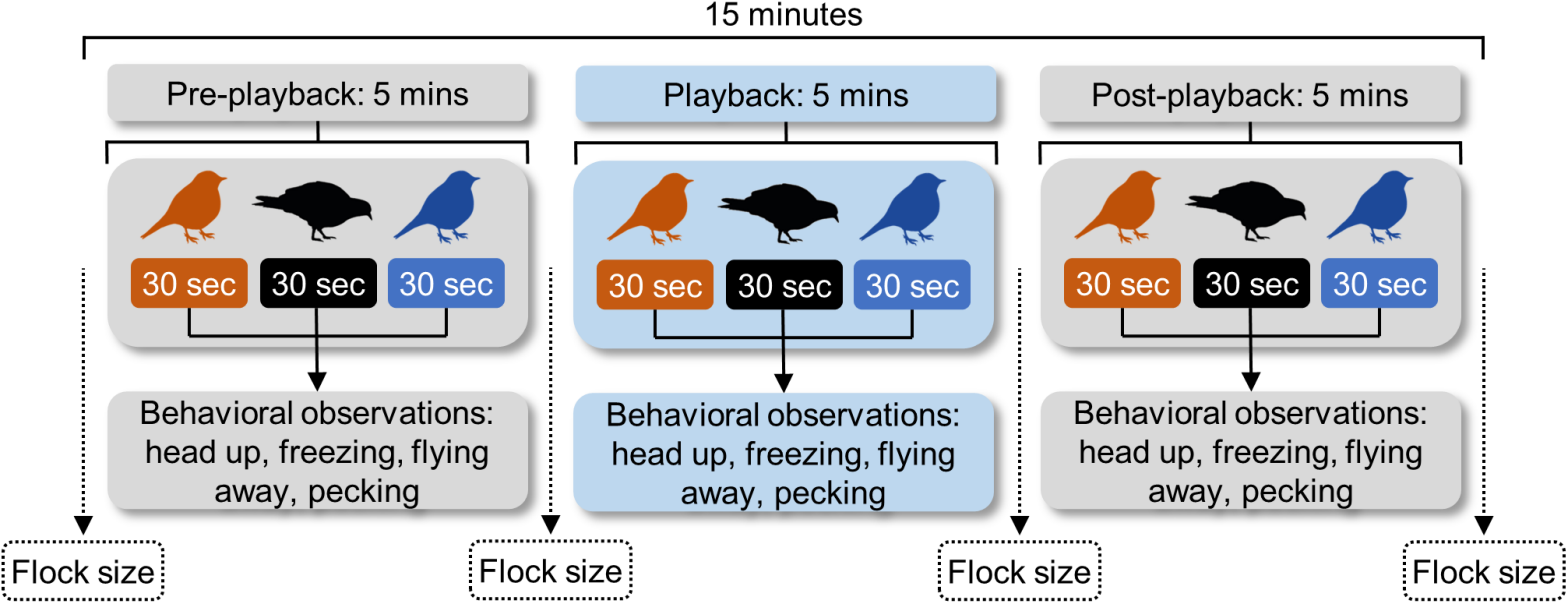
Representation of one experiment. During each period, observers made behavioral observations for 30 s on individual birds; observers counted flock size before and after each period.

While there are diversity of approaches for measuring antipredator behaviors in birds (e.g., decoys, flight initiation distance), we used an audio playback experiment using a Cooper's hawk call. A playback experiment allowed us to maintain a consistent predator cue that could be easily deployed by citizen scientists. Further, Cooper's hawk calls are simple in structure, stereotyped, and have been used previously for eliciting antipredator responses in passerines (Akçay et al., 2016; Pettinga et al., 2016; Schmidt et al., 2008). We sent FeederWatch volunteers a playback experiment kit that consisted of a digital voice recorder, tripod, and a remote‐controlled speaker (FoxPro) with pre‐loaded audio tracks, which emitted either a predator call (Cooper's hawk) or a control call of a nonthreatening songbird common in Chicago, the American goldfinch (*Spinus tristis*). We obtained representative vocalizations from the Macaulay Library at the Cornell Lab of Ornithology. Each audio track—hawk (predator) and goldfinch (control)—was 15 min long and consisted of three periods, as follows: (1) pre‐playback, 5 min of silence; (2) playback, 5 min alternating between 5 seconds of the call stimulus and 25 seconds of silence; and (3) post‐playback, 5 min of silence (Figure 2). We preset the speakers to broadcast tracks at 80 db SPL measured at 1 m (BAFX Products decibel meter; Akçay et al., 2016; Pettinga et al., 2016; Schmidt et al., 2008).

We instructed participants to place the speaker on the fully extended tripod (1.5 m) above ground level, near vegetative cover, and approximately 10 m from their feeder. We selected 10 m because it likely approaches the closest range at which a hawk would be perceived audibly by songbirds rather than visually (Pettinga et al., 2016). However, limited space in some backyards required that some speakers be placed closer than 10 m (mean = 9 m, min = 5 m, max = 12 m).

### 2.3 Data collection

The experiment consisted of two playback types (hawk and goldfinch) completed within a two‐day period, approximately twice a month. On the first day, the participant would flip a coin to decide which playback type to conduct first. On the second day, the participants started with the opposite playback type.

During each playback, participants recorded two forms of data: flock sizes and focal individual behavioral observations (Figure 2). Participants recorded flock size (the maximum number of each species seen at the feeder) before and after each period and focal behavioral data during each of the three periods (Figure 2). Although many factors influence an individual's vigilance in a flock (e.g., food quantity and quality, age and dominance, competition, and distance‐to‐neighbor; Beauchamp, 2008), flock size is thought to mediate individual responses to predation risk and is readily measured by citizen scientists.

We employed a focal‐switch observation approach (Losito et al., 1989). For focal behavioral data collection, participants watched an individual bird for approximately 30 s and recorded the bird's activities into a handheld voice recorder. Throughout the 5‐min observation period (15 min for the three observation periods), participants observed as many individuals as possible. If few birds were present, they repeated observations on the same individual. Participants recorded four focal behaviors: flying from feeder, freezing, head up, and pecking. Flight from the feeder was broken into three additional categories: flying within the feeder area, flying to cover within the feeder area, or flying away.

Participants submitted their voice‐recorded behavioral observations and flock size datasheets on a regular basis. We processed the voice‐recorded observations and transcribed—for each focal bird—the species, playback type (hawk or goldfinch), period it was observed (pre‐playback, playback, and post‐playback), number of each behavior, time of day to the nearest hour, and observation duration.

We were also interested in whether antipredator behaviors varied by (1) the presence of a sentinel species, (2) flock size, (3) body mass, (4) amount of protective cover near the feeder, and (5) level of urbanization surrounding the site. To evaluate the effect of sentinel species, we derived a binary variable indicating whether or not a black‐capped chickadee was present or absent or all periods within each playback experiment. Chickadees function as sentinels by producing antipredator mobbing calls that elicit strong responses in other species (Hurd, 1996; Turcotte & Desrochers, 2002). In calculating the presence of a sentinel species and flock size, we used the flock counts from before each playback period to characterize conditions at the start of each period. We obtained species‐specific body mass (grams) from *The Sibley Guide to Birds* (Sibley, 2000). Finally, we calculated percentages of canopy cover within 100 m of sites and impervious cover within 3 km of sites from The National Land Cover Database (Coulston et al., 2012; Homer et al., 2015; Jin et al., 2013; Song, 2005; Xian et al., 2011) as proxies for the amount of protective cover near feeders and the level of urbanization within the surrounding landscape, respectively.

### 2.4 Statistical analysis

Based on our hypotheses, we constructed 12 models to explore variation in antipredator behavior of feeder birds in relation to perceived predation risk, presence of a sentinel species, body mass, flock size, amount of protective cover, and amount of urbanization surrounding the sites (Table 1). We restricted analyses to species with at least 10 behavior observations. We then used model selection (Burnham & Anderson, 2002; Hurvich & Tsai, 1989) to determine which predictors best explained antipredator behavior. We fit the same candidate models in two separate analyses for (1) proportion of behaviors that were head up (hereafter “vigilant” models) and (2) proportion of behaviors that were pecking (hereafter “foraging” models) as response variables. We calculated the proportion of a given behavior as the number of times a bird displayed that behavior (e.g., had its head up) divided by the total number of behaviors that were counted for that individual during the observation period. Of the four behaviors recorded, head up (vigilance) and pecking (foraging) comprised 76% of the total behaviors observed. Moreover, there were few instances in which an individual flew from a feeder and returned during the duration of the observation. Consequently, the flying‐from‐feeder behavior was rarely counted multiple times per individual. Therefore, we restricted analysis to vigilance and foraging behaviors. However, we used all behaviors to calculate the proportion of vigilance and foraging.

**TABLE 1:** The 12 candidate models tested for the proportion of vigilant behavior

Model	ΔAIC_c_	*K*	*w*_*i*_
**Period × Playback × Flock size**	0	18	1
Period × Playback + Flock size	14.70	13	0
Period × Playback	16.44	13	0
Period × Playback + Mass	17.05	13	0
Period × Playback + Sentinel	18.35	13	0
Period × Playback + Impervious surface (3 km)	18.37	13	0
Period × Playback + Canopy cover (100 m)	18.43	13	0
Period × Playback × Mass	19.78	18	0
Period x Playback × Sentinel	21.32	18	0
Period × Playback × Impervious surface (3 km)	22.12	18	0
Period × Playback × Canopy cover (100 m)	21.86	18	0
Null	33.76	7	0

*Note*: All models—including the null—included control variables for time of day, temperature, year, and observation duration, as well as random effects for species and site. Bold indicates the top model. *K* = number of parameters and *w* = weight of evidence.

We fit generalized linear mixed‐effects models with a binomial distribution and logit link function for all models (Zuur et al., 2009). The dependent variable (proportion of either vigilance or foraging behavior) was weighted by the total number of behaviors observed during each focal bird observation. We fit models using the R package *lme4* (Bates et al., 2015). Every model—including the null model—contained predictors for time of day, daily mean temperature, year, and observation duration, as well as random effects for species (to control for interspecific differences) and site (to control for unmodeled variation among sites and participant). For all sites, we obtained daily mean temperature (°C) for Chicago's O'Hare airport from Weather Underground (https://www.wunderground.com/history). We standardized all continuous predictors by dividing their means by one standard deviation.

To test our hypotheses, we used a series of additive effects and two‐ and three‐way interactions between playback type (hawk or goldfinch), period, and each of the five focal predictors. Specifically, the model set contained: (1) one model with the two‐way interaction between playback type and period; (2) five models, each with the two‐way interaction and one additive effect of sentinel species, body mass, flock size, tree cover, or urbanization, (3) five models, each with a three‐way interaction between playback type, period, and one of the five predictors; and (4) a null model, containing only the control variables (Table 1).

We used AICc to rank models based on their ability to explain variance in the data, and used Akaike weights (*w*_*i*_) to estimate relative likelihood of each model given the data (Burnham & Anderson, 2002). We considered models with differences in AIC_*c*_ values (ΔAIC_*c*_) < 2.0 to be equivalent (Burnham & Anderson, 2002). We used the “Wald” method to calculate 95% confidence intervals around parameter estimates from the top model for each of the two model sets. Parameter estimates that did not overlap zero were considered significant. Lastly, for any top model containing the two‐way interaction between playback treatment and period, we ran multiple comparison *post‐hoc* Tukey tests to further explore the effect of playback treatment and period on feeder bird behavior.

## 3 RESULTS

During the 2016–2017 and 2017–2018 winter seasons, participants from 15 sites counted 1194 flocks and recorded behaviors from 3891 individual observations across 22 species; there were multiple observations per flock and occasionally per individual for cases in which there were no other birds available to be observed.

**FIGURE 3:**
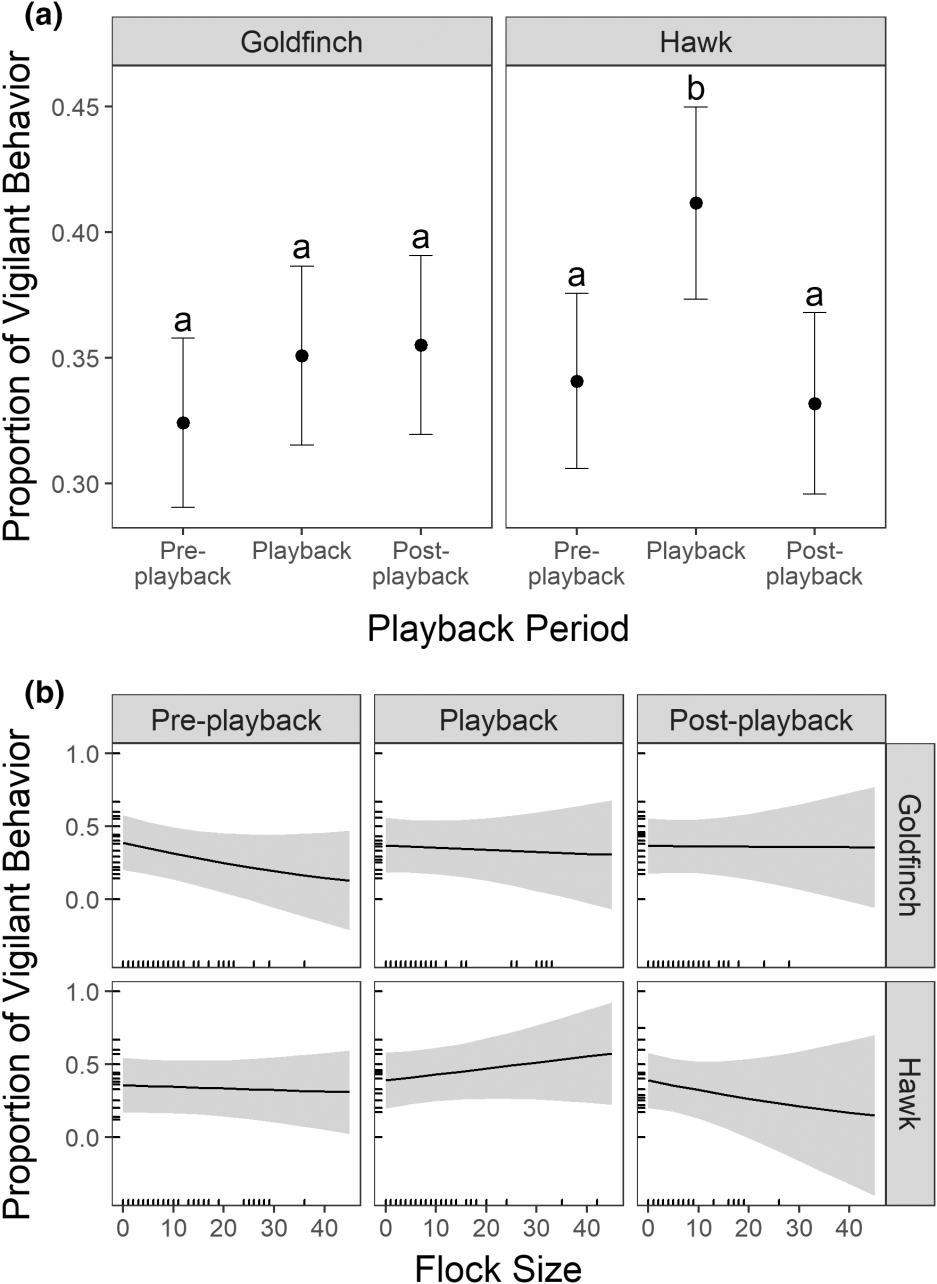
(a) Across all bird species, vigilance showed no change during the playback of the goldfinch call, but vigilance increased significantly during the hawk playback. (b) The increase in vigilance in response to the hawk playback was higher in larger flocks but declined significantly in larger flocks post‐playback. Error bars in (a) and gray ribbons in (b) represent the standard errors.

Across all playback periods, backyard birds showed greater vigilance later in the day and less vigilance during colder temperatures (Table S1). In addition, across all periods birds within larger flocks were generally less vigilant (Table S1), but this changed during the playback experiment (see below). The top‐ranked vigilance model contained the three‐way interaction between playback treatment, period, and flock size (*w* = 1.0, Table 1). Birds were more vigilant during the hawk playback than any other treatment–period combination (Figure 3a), and birds showed no difference in vigilance during the goldfinch call (Figure 3a). The effect of flock size varied across playback type and playback period, and birds within larger flocks were more vigilant during the hawk playback (Figure 3b). After playback, vigilant behavior dropped slightly below pre‐playback levels for the hawk call, especially for birds in larger flocks (Figure 3b), and showed no clear relationship with flock size for the goldfinch call (Figure 3b). We did not find effects of sentinel species or surrounding cover (impervious surface or tree canopy cover) on vigilance (Table 1).

**FIGURE 4:**
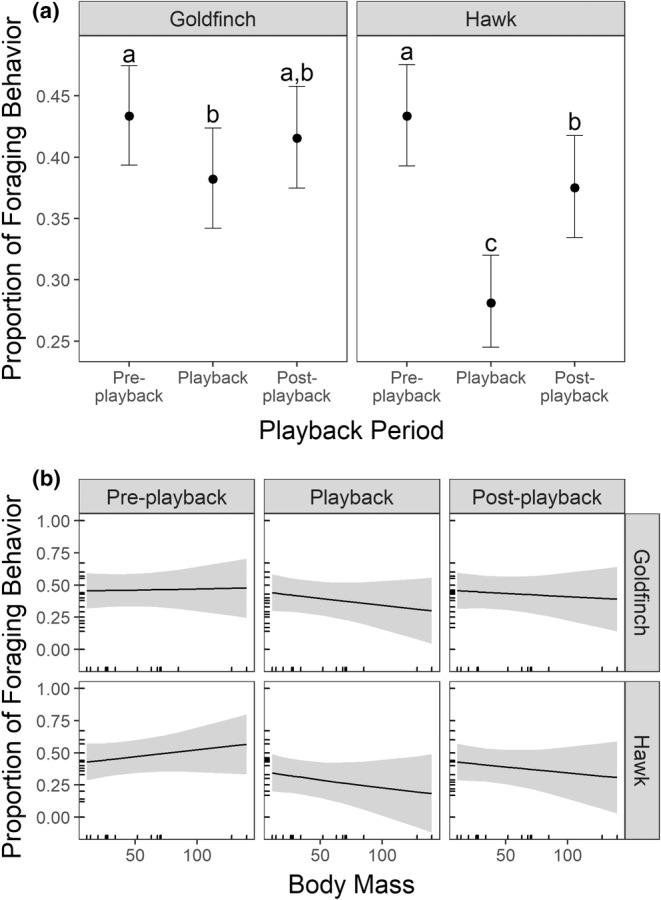
(a) Birds reduced their foraging behavior during the hawk playback period. (b) The reduction in foraging behavior was strongest for larger birds being exposed to hawk playback. Error bars in (a) and gray ribbons in (b) represent the standard errors.

The top‐ranked foraging model contained the three‐way interaction between playback type, period, and body mass (*w* = 0.99; Table 1, 2). We found strong support that the playback type and period interaction had an effect on foraging (Figure 4; Table 2). During the hawk call, birds spent less time foraging than any other playback type–period combination (Figure 4a). Birds were slightly less likely to forage during the goldfinch call compared with the pre‐playback period, but not significantly (Figure 4a). Finally, we found support for a two‐way interaction between body mass and playback period as larger‐bodied birds were less likely to forage during the playback and post‐playback periods, especially during hawk calls (Figure 4b). Similar to vigilance, there was a positive effect of observation duration on the proportion of foraging observed (Table S2). We did not find any support for the effects of sentinel species or surrounding cover (impervious surface or tree canopy cover) on foraging (Table 2)

**TABLE 2:** The 12 candidate models tested for the proportion of foraging behavior

Model	ΔAIC_c_	*K*	*w*_*i*_
**Period × Playback × Mass**	0	18	0.99
Period × Playback × Flock size	9.47	18	0.01
Period × Playback + Flock size	13.56	13	0
Period × Playback × Canopy cover (100 m)	16.56	18	0
Period × Playback	18.03	12	0
Period × Playback × Impervious surface (3 km)	19.35	18	0
Period × Playback + Mass	19.45	13	0
Period × Playback + Impervious surface (3 km)	19.73	13	0
Period × Playback + Sentinel	19.91	13	0
Period x Playback × Sentinel	21.71	18	0
Period × Playback + Canopy cover (100 m)	67.26	12	0
Null	89.75	7	0

*Note*: All models—including the null—included control variables for time of day, temperature, year, and observation duration, as well as random effects for species and site. Bold indicates the top model. *K* = number of parameters and *w* = weight of evidence.

## 4 DISCUSSION

Our goal was to demonstrate the potential for citizen scientists to deploy an experiment focused on the antipredator behavior of backyard birds. By engaging participants from an established citizen science program (Project FeederWatch), we experimentally explored bird antipredator behaviors in response to cues from a native predator that has been rapidly colonizing urban and suburban landscapes throughout North America. Citizen scientists collected nearly 4000 individual antipredator behaviors across 22 bird species within an urban environment. Generally, our broader hypothesis that backyard birds in human‐modified landscapes maintained antipredator behaviors was supported; backyard birds were more vigilant and foraged less during the hawk playback and showed little to no response to the goldfinch (control) call. Despite evidence from other studies that urban birds showed higher tolerance to predation risk compared with their rural counterparts (Møller et al., 2013; Samia et al., 2015), our behavioral experiment indicates that many of these critical antipredator behaviors persist.

Many of the backyard birds in our playback experiment displayed increased vigilance and decreased foraging in response to the hawk call, but these responses varied across flock and body sizes. While we had expected that individual birds in larger flocks would be less responsive to a predator cue due to a group size effect (Beauchamp, 2008; Elgar, 1989; Xu et al., 2013), we found that birds within larger flocks were *more* vigilant during the hawk playback. An increase in vigilance for individuals within larger flocks may suggest that individuals mimic the vigilance behaviors of their flockmates. We did, however, detect slightly less vigilance in larger flocks after the hawk playback, suggesting that the group size effect may allow individual birds to more quickly resume typical behavior following exposure to a predator. Body size is considered a strong predictor of species vulnerability to disturbance, and larger‐bodied birds generally detect potential predators at greater distances and are more responsive to disturbance (Bennett & Owens, 2002; Blumstein, 2014). In our experiment, larger‐bodied species were more responsive and spent significantly less time foraging during the hawk playback. Cooper's hawks primarily prey on such larger‐bodied birds; in studies of urban hawk diets, the majority of prey consumed were European starlings, mourning doves (*Zenaida macroura*), and rock pigeons (*Columba livia*; Cava et al., 2012; Roth II & Lima, 2003). Consequently, it would appear that these larger‐bodied birds are more sensitive to a predator cue. Notably, for both vigilance and foraging, we did not find compelling evidence that antipredator behaviors were mediated by the presence of a sentinel species, canopy cover, or urbanization levels.

Citizen science has become an essential ecological research tool of the 21st century (Cooper et al., 2014). The biological data collected by citizen scientists has opened new avenues of scientific study, but citizen science typically entails the collection of observational data. There are good reasons for an emphasis on observational data, as citizen science programs must balance recruiting a large number of participants (often thousands or more) with establishing sampling protocols and technologies that are efficient and easy to use. However, experimental ecology offers the potential to isolate causes underpinning behavioral outcomes by applying treatments under similar conditions (Cooke et al., 2017).

Our study demonstrates that citizen scientists are capable of conducting ecological experiments, but it is clear that the protocols must be efficient, repeatable, and easy to implement. Many citizen science programs are considered big data initiatives that must account for data volume, velocity, and variety (Bonter & Cooper, 2012; La Sorte et al., 2018). Citizen‐science programs spend considerable time designing collection protocols and adapting technological advancements (e.g., smartphones, online crowdsourcing) to increase data volume and engage new audiences (Newman et al., 2012). Different from more traditional programs, citizen‐science experimentation has unique challenges. Our playback experiment required sending specialized equipment to participants, and we were compelled to simplify protocols upon receiving input from participants. In addition, our reliance on an established citizen science program may have restricted our ability to reach communities that are historically under‐represented in citizen science (Pandya, 2012). Although data volume is less of a concern for experimentation due to the engagement of fewer participants, the processing of auditory data and classification was time‐intensive and could limit scalability. Finally, the granularity of the response data (e.g., behavioral data vs. species occurrences) likely introduces new sources of observation bias and misinterpretations that are difficult to assess and fully evaluate.

An important aspect of this study is the engagement of citizen scientists in urban ecology. Projections suggest that 68% of the world's population will live in urban areas by 2050 (United Nations, 2019) and urbanized land is increasing more quickly than all other land cover types (Pickett et al., 2011), yet urban audiences are underserved by most existing citizen science programs (Cooper et al., 2007). Urban‐focused research is underrepresented in ecology (just 0.4–6% of the ecology literature; Collins et al., 2000; Martin et al., 2012; Miller & Hobbs, 2002), but the use of citizen science experimentation offers a unique platform for increasing scientific literacy and education while increasing ecological knowledge in urban settings. Finally, expanding citizen science experimentation may confer broader societal benefits, since participating in citizen science often increases participants' engagement and trust in science (Fernandez‐Gimenez et al., 2008; Lewandowski & Oberhauser, 2017) and improves their sense of well‐being (Bell et al., 2008; Schuttler et al., 2018).

## AUTHOR CONTRIBUTIONS

**Benjamin Zuckerberg:** Conceptualization (equal); data curation (supporting); formal analysis (supporting); funding acquisition (lead); investigation (equal); methodology (equal); project administration (equal); resources (equal); software (equal); supervision (lead); validation (equal); visualization (supporting); writing – original draft (lead); writing – review and editing (equal). **Jennifer D. McCabe:** Conceptualization (equal); data curation (lead); formal analysis (lead); funding acquisition (supporting); investigation (equal); methodology (lead); project administration (supporting); resources (equal); software (equal); supervision (supporting); validation (equal); visualization (equal); writing – original draft (supporting); writing – review and editing (equal). **Neil A Gilbert:** Conceptualization (supporting); data curation (supporting); formal analysis (supporting); funding acquisition (supporting); investigation (supporting); methodology (supporting); project administration (supporting); resources (supporting); software (supporting); supervision (supporting); validation (supporting); visualization (lead); writing – original draft (supporting); writing – review and editing (equal).

## CONFLICT OF INTEREST

We have no conflict of interests to report.

## FUNDING INFORMATION

This project was funded by NASA's Citizen Science for Earth Systems Program (NNX17AI68A). We acknowledge additional support from the Department of Forest and Wildlife Ecology, University of Wisconsin‐Madison.

## Supporting information


Appendix S1

## Data Availability

Data have been uploaded to Dryad. DOI accession number: https://doi.org/10.5061/dryad.tmpg4f521.

## References

[ece39269-bib-0001] Acuto, M. , & Parnell, S. (2016). Leave no city behind. Science, 352(6288), 873. 10.1126/science.aag1385 27199390

[ece39269-bib-0002] Akçay, Ç. , Clay, A. , Campbell, S. E. , & Beecher, M. D. (2016). The sparrow and the hawk: Aggressive signaling under risk of predation. Behavioral Ecology, 27(2), 601–607. 10.1093/beheco/arv196

[ece39269-bib-0003] Bates, D. , Mächler, M. , Bolker, B. , & Walker, S. (2015). Fitting linear mixed‐effects models using lme4. Journal of Statistical Software, 67(1), 1–48. 10.18637/jss.v067.i01

[ece39269-bib-0004] Beauchamp, G. (2003). Group‐size effects on vigilance: A search for mechanisms. Behavioural Processes, 63(3), 141–145. 10.1016/S0376-6357(03)00011-1 12829314

[ece39269-bib-0005] Beauchamp, G. (2008). What is the magnitude of the group‐size effect on vigilance? Behavioral Ecology, 19(6), 1361–1368. 10.1093/beheco/arn096

[ece39269-bib-0006] Becker, D. J. , & Hall, R. J. (2014). Too much of a good thing: Resource provisioning alters infectious disease dynamics in wildlife. Biology Letters, 10(7), 20140309. 10.1098/rsbl.2014.0309 25055815 PMC4126624

[ece39269-bib-0007] Bell, S. , Marzano, M. , Cent, J. , Kobierska, H. , Podjed, D. , Vandzinskaite, D. , Reinert, H. , Armaitiene, A. , Grodzińska‐Jurczak, M. , & Muršič, R. (2008). What counts? Volunteers and their organisations in the recording and monitoring of biodiversity. Biodiversity and Conservation, 17(14), 3443–3454. 10.1007/s10531-008-9357-9

[ece39269-bib-0008] Bennett, P. , & Owens, I. (2002). Evolutionary ecology of birds: Life histories, mating systems, and extinction. Oxford University Press.

[ece39269-bib-0009] Blumstein, D. (2014). Attention, habituation, and antipredator behaviour: Implications for urban birds. In D. Gill & H. Brumm (Eds.), Avian urban ecology: Behavioural and physiological adaptations (pp. 41–53). Oxford University Press.

[ece39269-bib-0010] Boal, C. , & Dykstra, C. (2018). Urban raptors: Ecology and conservation of birds of prey in cities. Island Press.

[ece39269-bib-0011] Bonter, D. N. , & Cooper, C. B. (2012). Data validation in citizen science: A case study from Project FeederWatch. Frontiers in Ecology and the Environment, 10(6), 305–307. 10.1890/110273

[ece39269-bib-0012] Brown, J. S. , Laundré, J. W. , & Gurung, M. (1999). The ecology of fear: Optimal foraging, game theory, and trophic interactions. Journal of Mammalogy, 80(2), 385–399. 10.2307/1383287

[ece39269-bib-0013] Burnham, K. , & Anderson, D. (2002). Model selection and multimodel inference: A practical information‐theoretic approach (2nd ed.). Springer Verlag.

[ece39269-bib-0014] Carthey, A. J. R. , & Blumstein, D. T. (2018). Predicting predator recognition in a changing world. Trends in Ecology & Evolution, 33(2), 106–115. 10.1016/j.tree.2017.10.009 29132776

[ece39269-bib-0015] Cava, J. , Stewart, A. , & Rosenfield, R. (2012). Introduced species dominate the diet of breeding urban Cooper's Hawks in British Columbia. The Wilson Journal of Ornithology, 124(4), 775–782.

[ece39269-bib-0016] Collins, J. P. , Kinzig, A. , Grimm, N. B. , Fagan, W. F. , Hope, D. , Wu, J. , & Borer, E. T. (2000). A New Urban Ecology: Modeling human communities as integral parts of ecosystems poses special problems for the development and testing of ecological theory. American Scientist, 88(5), 416–425.

[ece39269-bib-0017] Cooke, S. J. , Birnie‐Gauvin, K. , Lennox, R. J. , Taylor, J. J. , Rytwinski, T. , Rummer, J. L. , Franklin, C. E. , Bennett, J. R. , & Haddaway, N. R. (2017). How experimental biology and ecology can support evidence‐based decision‐making in conservation: Avoiding pitfalls and enabling application. Conservation Physiology, 5(1), 1–14. 10.1093/conphys/cox043 PMC5550808 28835842

[ece39269-bib-0018] Cooper, C. (2016). Citizen science: How ordinary people are changing the face of discovery. Abrams.

[ece39269-bib-0019] Cooper, C. , Dickinson, J. , Phillips, T. , & Bonney, R. (2007). Citizen science as a tool for conservation in residential ecosystems. Ecology and Society, 12(2), 11. 10.5751/ES-02197-120211

[ece39269-bib-0020] Cooper, C. , Shirk, J. , & Zuckerberg, B. (2014). The invisible prevalence of citizen science in global research: Migratory birds and climate change. PLoS One, 9(9), e106508. 10.1371/journal.pone.0106508 25184755 PMC4153593

[ece39269-bib-0021] Cooper, W. E. , & Wilson, D. S. (2007). Beyond optimal escape theory: Microhabitats as well as predation risk affect escape and refuge use by the phrynosomatid lizard Sceloporus virgatus. Behaviour, 144(10), 1235–1254.

[ece39269-bib-0022] Coulston, J. W. , Moisen, G. G. , Wilson, B. T. , Finco, M. V. , Cohen, W. B. , & Brewer, C. K. (2012). Modeling percent tree canopy cover: A pilot study. Photogrammetric Engineering & Remote Sensing, 78(7), 715–727.

[ece39269-bib-0023] Díaz, M. , Møller, A. P. , Flensted‐Jensen, E. , Grim, T. , Ibáñez‐Álamo, J. D. , Jokimäki, J. , Markó, G. , & Tryjanowski, P. (2013). The geography of fear: A latitudinal gradient in anti‐predator escape distances of birds across Europe. PLoS One, 8(5), e64634. 10.1371/journal.pone.0064634 23724070 PMC3665823

[ece39269-bib-0024] Dickinson, J. L. , Zuckerberg, B. , & Bonter, D. N. (2010). Citizen science as an ecological research tool: Challenges and benefits. Annual Review of Ecology, Evolution, and Systematics, 41(1), 149–172. 10.1146/annurev-ecolsys-102209-144636

[ece39269-bib-0025] Elgar, M. (1989). Predator vigilance and group size in mammals and birds: A critical review of the empirical evidence. Biological Reviews, 64, 13–33. 2655726 10.1111/j.1469-185x.1989.tb00636.x

[ece39269-bib-0026] Estes, J. , Terborgh, J. , Brashares, J. S. , Power, M. E. , Berger, J. , Bond, W. J. , Carpenter, S. R. , Essington, T. E. , Holt, R. D. , Jackson, J. B. C. , Marquis, R. J. , Oksanen, L. , Oksanen, T. , Paine, R. T. , Pikitch, E. K. , Ripple, W. J. , Sandin, S. A. , Scheffer, M. , Schoener, T. W. , … Wardle, D. A. (2011). Trophic downgrading of planet earth. Science, 333(6040), 301–306. 10.1126/science.1205106 21764740

[ece39269-bib-0027] Estes, W. , & Mannan, R. W. (2003). Feeding behavior of Cooper's Hawks at urban and rural nests in southeastern Arizona. The Condor, 105(1), 107–116. 10.1093/condor/105.1.107

[ece39269-bib-0028] Fernandez‐Gimenez, M. , Ballard, H. , & Sturtevant, V. (2008). Adaptive management and social learning in collaborative and community‐based monitoring: A study of five community‐based forestry organizations in the western USA. Ecology and Society, 13(2), 4. 10.5751/ES-02400-130204

[ece39269-bib-0029] Fischer, J. D. , Cleeton, S. H. , Lyons, T. P. , & Miller, J. R. (2012). Urbanization and the predation paradox: The role of trophic dynamics in structuring vertebrate communities. Bioscience, 62(9), 809–818. 10.1525/bio.2012.62.9.6

[ece39269-bib-0030] Fraser, L. H. , Henry, H. A. , Carlyle, C. N. , White, S. R. , Beierkuhnlein, C. , Cahill, J. F. , Casper, B. B. , Cleland, E. , Collins, S. L. , Dukes, J. S. , Knapp, A. K. , Lind, E. , Long, R. , Luo, Y. , Reich, P. B. , Smith, M. D. , Sternberg, M. , & Turkington, R. (2013). Coordinated distributed experiments: An emerging tool for testing global hypotheses in ecology and environmental science. Frontiers in Ecology and the Environment, 11(3), 147–155. 10.1890/110279

[ece39269-bib-0031] Galbraith, J. A. , Beggs, J. R. , Jones, D. N. , & Stanley, M. C. (2015). Supplementary feeding restructures urban bird communities. Proceedings of the National Academy of Sciences, 112(20), E2648–E2657. 10.1073/pnas.1501489112 PMC4443315 25941361

[ece39269-bib-0032] Gao, J. , & O'Neill, B. C. (2020). Mapping global urban land for the 21st century with data‐driven simulations and Shared Socioeconomic Pathways. Nature Communications, 11(1), 2302. 10.1038/s41467-020-15788-7 PMC7210308 32385275

[ece39269-bib-0033] Gaynor, K. M. , Brown, J. S. , Middleton, A. D. , Power, M. E. , & Brashares, J. S. (2019). Landscapes of fear: Spatial patterns of risk perception and response. Trends in Ecology & Evolution, 34(4), 355–368. 10.1016/j.tree.2019.01.004 30745252

[ece39269-bib-0034] Gracanin, A. , Roger, E. , Katsis, A. C. , O'Loughlin, L. S. , Emery, N. J. , Ocock, J. F. , & O'Hanlon, J. C. (2020). An artificial bird nest experiment in urban environments: Lessons from a school‐based citizen science programme. Austral Ecology, 45(5), 523–528. 10.1111/aec.12859

[ece39269-bib-0035] Griesser, M. , & Nystrand, M. (2009). Vigilance and predation of a forest‐living bird species depend on large‐scale habitat structure. Behavioral Ecology, 20(4), 709–715. 10.1093/beheco/arp045

[ece39269-bib-0036] Guiden, P. W. , Bartel, S. L. , Byer, N. W. , Shipley, A. A. , & Orrock, J. L. (2019). Predator–prey interactions in the anthropocene: Reconciling multiple aspects of novelty. Trends in Ecology & Evolution, 34(7), 616–627. 10.1016/j.tree.2019.02.017 30902358

[ece39269-bib-0037] Homer, C. , Dewitz, J. , Yang, L. , Jin, S. , Danielson, P. , Xian, G. , Coulston, J. , Herold, N. , Wickham, J. , & Megown, K. (2015). Completion of the 2011 national land cover database for the conterminous United States—representing a decade of land cover change information. Photogrammetric Engineering and Remote Sensing, 81(5), 345–354. 10.14358/PERS.81.5.345

[ece39269-bib-0038] Hurd, C. R. (1996). Interspecific attraction to the mobbing calls of black‐capped chickadees (*Parus atricapillus*). Behavioral Ecology and Sociobiology, 38(4), 287–292. 10.1007/s002650050244

[ece39269-bib-0039] Hurvich, C. , & Tsai, C. (1989). Regression and time series model selection in small samples. Biometrika, 76(2), 297–307. 10.1093/biomet/76.2.297

[ece39269-bib-0040] Jin, S. , Yang, L. , Danielson, P. , Homer, C. , Fry, J. , & Xian, G. (2013). A comprehensive change detection method for updating the National Land Cover Database to circa 2011. Remote Sensing of Environment, 132, 159–175. 10.1016/j.rse.2013.01.012

[ece39269-bib-0041] Kaartinen, R. , Hardwick, B. , & Roslin, T. (2013). Using citizen scientists to measure an ecosystem service nationwide. Ecology, 94(11), 2645–2652. 10.1890/12-1165.1 24400516

[ece39269-bib-0042] La Sorte, F. A. , Lepczyk, C. A. , Burnett, J. L. , Hurlbert, A. H. , Tingley, M. W. , & Zuckerberg, B. (2018). Opportunities and challenges for big data ornithology. The Condor, 120(2), 414–426. 10.1650/CONDOR-17-206.1

[ece39269-bib-0043] Lewandowski, E. J. , & Oberhauser, K. S. (2017). Butterfly citizen scientists in the United States increase their engagement in conservation. Biological Conservation, 208, 106–112. 10.1016/j.biocon.2015.07.029

[ece39269-bib-0044] Lilly, M. V. , Lucore, E. C. , & Tarvin, K. A. (2019). Eavesdropping grey squirrels infer safety from bird chatter. PLoS One, 14(9), e0221279. 10.1371/journal.pone.0221279 31483829 PMC6726132

[ece39269-bib-0045] Lima, S. L. (1987). Vigilance while feeding and its relation to the risk of predation. Journal of Theoretical Biology, 124(3), 303–316. 10.1016/S0022-5193(87)80118-2

[ece39269-bib-0046] Lima, S. L. , & Dill, L. M. (1990). Behavioral decisions made under the risk of predation: A review and prospectus. Canadian Journal of Zoology, 68(4), 619–640. 10.1139/z90-092

[ece39269-bib-0047] Losito, M. , Mirarchi, R. , & Baldassarre, G. (1989). New techniques for time‐activity studies of avian flocks in view‐restricted habitats. Journal of Field Ornithology, 60(3), 388–396.

[ece39269-bib-0048] Manlick, P. J. , & Pauli, J. N. (2020). Human disturbance increases trophic niche overlap in terrestrial carnivore communities. Proceedings of the National Academy of Sciences, 117(43), 26842–26848. 10.1073/pnas.2012774117 PMC7604423 33046630

[ece39269-bib-0049] Martin, L. J. , Blossey, B. , & Ellis, E. (2012). Mapping where ecologists work: Biases in the global distribution of terrestrial ecological observations. Frontiers in Ecology and the Environment, 10(4), 195–201. 10.1890/110154

[ece39269-bib-0050] McCabe, J. D. , Yin, H. , Cruz, J. , Radeloff, V. , Pidgeon, A. , Bonter, D. N. , & Zuckerberg, B. (2018). Prey abundance and urbanization influence the establishment of avian predators in a metropolitan landscape. Proceedings of the Royal Society B: Biological Sciences, 285(1890), 20182120. 10.1098/rspb.2018.2120 PMC6235051 30404886

[ece39269-bib-0051] Miller, J. R. , & Hobbs, R. J. (2002). Conservation where people live and work. Conservation Biology, 16(2), 330–337. 10.1046/j.1523-1739.2002.00420.x

[ece39269-bib-0052] Mitchell, M. G. E. , Wu, D. , Johansen, K. , Maron, M. , McAlpine, C. , & Rhodes, J. R. (2016). Landscape structure influences urban vegetation vertical structure. Journal of Applied Ecology, 53(5), 1477–1488. 10.1111/1365-2664.12741

[ece39269-bib-0053] Moll, R. J. , Cepek, J. D. , Lorch, P. D. , Dennis, P. M. , Tans, E. , Robison, T. , Millspaugh, J. J. , & Montgomery, R. A. (2019). What does urbanization actually mean? A framework for urban metrics in wildlife research. Journal of Applied Ecology, 56(5), 1289–1300. 10.1111/1365-2664.13358

[ece39269-bib-0054] Møller, A. P. (2008). Flight distance of urban birds, predation, and selection for urban life. Behavioral Ecology and Sociobiology, 63(1), 63–75. 10.1007/s00265-008-0636-y

[ece39269-bib-0055] Møller, A. P. , Díaz, M. , Flensted‐Jensen, E. , Grim, T. , Ibáñez‐Álamo, J. D. , Jokimäki, J. , Mänd, R. , Markó, G. , & Tryjanowski, P. (2015). Urbanized birds have superior establishment success in novel environments. Oecologia, 178(3), 943–950. 10.1007/s00442-015-3268-8 25694044

[ece39269-bib-0056] Møller, A. P. , Grim, T. , Ibáñez‐Álamo, J. D. , Markó, G. , & Tryjanowski, P. (2013). Change in flight initiation distance between urban and rural habitats following a cold winter. Behavioral Ecology, 24(5), 1211–1217. 10.1093/beheco/art054

[ece39269-bib-0057] Newman, G. , Wiggins, A. , Crall, A. , Graham, E. , Newman, S. , & Crowston, K. (2012). The future of citizen science: Emerging technologies and shifting paradigms. Frontiers in Ecology and the Environment, 10(6), 298–304. 10.1890/110294

[ece39269-bib-0058] Newsome, T. M. , Dellinger, J. A. , Pavey, C. R. , Ripple, W. J. , Shores, C. R. , Wirsing, A. J. , & Dickman, C. R. (2015). The ecological effects of providing resource subsidies to predators. Global Ecology and Biogeography, 24(1), 1–11. 10.1111/geb.12236

[ece39269-bib-0059] Oro, D. , Genovart, M. , Tavecchia, G. , Fowler, M. S. , & Martínez‐Abraín, A. (2013). Ecological and evolutionary implications of food subsidies from humans. Ecology Letters, 16(12), 1501–1514. 10.1111/ele.12187 24134225

[ece39269-bib-0060] Pandya, R. E. (2012). A framework for engaging diverse communities in citizen science in the US. Frontiers in Ecology and the Environment, 10(6), 314–317. 10.1890/120007

[ece39269-bib-0061] Pettinga, D. , Kennedy, J. , & Proppe, D. S. (2016). Common urban birds continue to perceive predator calls that are overlapped by road noise. Urban Ecosystem, 19(1), 373–382. 10.1007/s11252-015-0498-9

[ece39269-bib-0062] Pickett, S. T. A. , Cadenasso, M. L. , Grove, J. M. , Boone, C. G. , Groffman, P. M. , Irwin, E. , Kaushal, S. S. , Marshall, V. , McGrath, B. P. , Nilon, C. H. , Pouyat, R. V. , Szlavecz, K. , Troy, A. , & Warren, P. (2011). Urban ecological systems: Scientific foundations and a decade of progress. Journal of Environmental Management, 92(3), 331–362. 10.1016/j.jenvman.2010.08.022 20965643

[ece39269-bib-0063] Pulliam, H. R. (1973). On the advantages of flocking. Journal of Theoretical Biology, 38(2), 419–422. 10.1016/0022-5193(73)90184-7 4734745

[ece39269-bib-0064] Roberts, G. (1996). Why individual vigilance declines as group size increases. Animal Behaviour, 51(5), 1077–1086. 10.1006/anbe.1996.0109

[ece39269-bib-0065] Rosenfield, R. (2018). The Cooper's Hawk: Breeding ecology and natural history of a winged huntsman. Hancock House.

[ece39269-bib-0066] Rosenfield, R. , Bielefeldt, J. , Gehring, J. , & Beckmann, D. (1995). Nesting density, nest area reoccupancy, and monitoring implications for Cooper's Hawks in Wisconsin. Journal of Raptor Research, 29, 1–4.

[ece39269-bib-0067] Rosenfield, R. , Madden, K. , Bielefeldt, J. , & Curtis, O. (2020). Cooper's Hawk (Accipiter cooperii), version 1.0. In P. Rodewald (Ed.), Birds of the World. Cornell Lab of Ornithology. 10.2173/bow.coohaw.01

[ece39269-bib-0068] Roth, T. C., II , & Lima, S. L. (2003). Hunting behavior and diet of Cooper's Hawks: An urban view of the small‐bird‐in‐winter paradigm. The Condor, 105(3), 474–483. 10.1093/condor/105.3.474

[ece39269-bib-0069] Samia, D. S. M. , Nakagawa, S. , Nomura, F. , Rangel, T. F. , & Blumstein, D. T. (2015). Increased tolerance to humans among disturbed wildlife. Nature Communications, 6(1), 8877. 10.1038/ncomms9877 PMC4660219 26568451

[ece39269-bib-0070] Schmidt, R. , Kunc, H. P. , Amrhein, V. , & Naguib, M. (2008). Aggressive responses to broadband trills are related to subsequent pairing success in nightingales. Behavioral Ecology, 19(3), 635–641. 10.1093/beheco/arn021

[ece39269-bib-0071] Schuttler, S. G. , Sorensen, A. E. , Jordan, R. C. , Cooper, C. , & Shwartz, A. (2018). Bridging the nature gap: Can citizen science reverse the extinction of experience? Frontiers in Ecology and the Environment, 16(7), 405–411. 10.1002/fee.1826

[ece39269-bib-0072] Seto, K. C. , Sánchez‐Rodríguez, R. , & Fragkias, M. (2010). The new geography of contemporary urbanization and the environment. Annual Review of Environment and Resources, 35(1), 167–194. 10.1146/annurev-environ-100809-125336

[ece39269-bib-0073] Shettleworth, S. (2009). Cognition, evolution, and behavior (2nd ed.). Oxford University Press.

[ece39269-bib-0074] Sibley, D. (2000). The Sibley Guide to Birds (1st ed.). Alfred A. Knopf.

[ece39269-bib-0075] Smith, J. A. , Suraci, J. P. , Hunter, J. S. , Gaynor, K. M. , Keller, C. B. , Palmer, M. S. , Atkins, J. L. , Castañeda, I. , Cherry, M. J. , Garvey, P. M. , Huebner, S. E. , Morin, D. J. , Teckentrup, L. , Weterings, M. J. A. , & Beaudrot, L. (2020). Zooming in on mechanistic predator–prey ecology: Integrating camera traps with experimental methods to reveal the drivers of ecological interactions. Journal of Animal Ecology, 89(9), 1997–2012. 10.1111/1365-2656.13264 32441766

[ece39269-bib-0076] Song, C. (2005). Spectral mixture analysis for subpixel vegetation fractions in the urban environment: How to incorporate endmember variability? Remote Sensing of Environment, 95(2), 248–263. 10.1016/j.rse.2005.01.002

[ece39269-bib-0077] Sullivan, B. L. , Wood, C. L. , Iliff, M. J. , Bonney, R. E. , Fink, D. , & Kelling, S. (2009). eBird: A citizen‐based bird observation network in the biological sciences. Biological Conservation, 142(10), 2282–2292. 10.1016/j.biocon.2009.05.006

[ece39269-bib-0078] Templeton, C. N. , & Greene, E. (2007). Nuthatches eavesdrop on variations in heterospecific chickadee mobbing alarm calls. Proceedings of the National Academy of Sciences, 104(13), 5479–5482. 10.1073/pnas.0605183104 PMC1838489 17372225

[ece39269-bib-0079] Turcotte, Y. , & Desrochers, A. (2002). Playbacks of mobbing calls of Black‐capped Chickadees help estimate the abundance of forest birds in winter. Journal of Field Ornithology, 73(3), 303–307. 10.1648/0273-8570-73.3.303

[ece39269-bib-0080] Uchida, K. , & Blumstein, D. T. (2021). Habituation or sensitization? Long‐term responses of yellow‐bellied marmots to human disturbance. Behavioral Ecology, 32, 668–678. 10.1093/beheco/arab016

[ece39269-bib-0081] United Nations . (2019). World Urbanization Prospects: The 2018 Revision (ST/ESA/SER.A/420). United Nations, Department of Economic and Social Affairs, Population Division.

[ece39269-bib-0082] Ware, H. E. , McClure, C. J. W. , Carlisle, J. D. , & Barber, J. R. (2015). A phantom road experiment reveals traffic noise is an invisible source of habitat degradation. Proceedings of the National Academy of Sciences, 112(39), 12105–12109. 10.1073/pnas.1504710112 PMC4593122 26324924

[ece39269-bib-0083] Wells, J. , Rosenberg, K. , Dunn, E. , Tessaglia‐Hymes, D. , & Dhondt, A. (1998). Feeder counts as indicators of spatial and temporal variation in winter abundance of resident birds. Journal of Field Ornithology, 69(4), 577–586.

[ece39269-bib-0084] Xian, G. Z. , Homer, C. G. , Dewitz, J. , Fry, J. , Hossain, N. , & Wickham, J. (2011). Change of impervious surface area between 2001 and 2006 in the conterminous United States. Photogrammetric Engineering and Remote Sensing, 77(8), 5.

[ece39269-bib-0085] Xu, F. , Ma, M. , Yang, W. , David, B. , Ding, P. , & Zhang, T. (2013). Vigilance in black‐necked cranes: Effects of predation vulnerability and flock size. Wilson Journal of Ornithology, 125, 208–212. 10.2307/41932857

[ece39269-bib-0086] Zuur, A. , Ieno, E. N. , Walker, N. , Saveliev, A. A. , & Smith, G. M. (2009). Mixed effects models and extensions in ecology with R. Springer‐Verlag. 10.1007/978-0-387-87458-6

