# Supplementary material for: Antipredator behaviors in urban settings: Ecological experimentation powered by citizen science
Source: Ecol Evol. 2022 Sep 9;12(9):e9269. doi: 10.1002/ece3.9269 (PMC9461346; doi:10.1002/ece3.9269)
Supplement: Supplementary file 1 — Appendix S1 [file ECE3-12-e9269-s001.docx]

To detect whether over- or under-dispersion occurred in our models, we estimated the dispersion parameter, ĉ, with the ratio of the deviance over the residual degrees of freedom (McCullagh & Nelder 1989) for all models. Values of ĉ approximating 1 indicate no over- or under-dispersion and, in general, a ĉ > 1.5 suggests inadequate fit (Zuur et al. 2009). If found, quasi-likelihood theory can be applied, modifying the Akaike’s Information Criteria (AIC) value associated with each model (Dingemanse et al. 2004). Model selection is then based on the quasi-AIC (QAIC) (Richards 2007).

We did not detect over- or under-dispersion in any of our model sets. Dispersion parameters ranged from 0.89 – 0.90, and 0.96 – 0.97 for our foraging and vigilant models, respectively.

Table S1: Coefficient means and 95% Confidence Intervals (CIs) for the model evaluating vigilance behavior of feeder birds in Chicago, IL. Estimates for categorical variables and interactions were calculated against the reference listed.

| Parameter | Mean | 2.5% CI | 97.5% CI |
| --- | --- | --- | --- |
| Pre-playback Period | -0.75 | -1.11 | -0.39 |
| Playback Period | -0.63 | -1.14 | -0.84 |
| Post-playback Period | -0.61 | -1.12 | 1.13 |
| Control | reference |  |  |
| Treatment | 0.07 | -0.07 | 0.22 |
| Time of Day | 0.07 | 0.02 | 0.12 |
| Min. Temperature | -0.07 | -0.13 | 0.01 |
| Duration | 0.25 | 0.21 | 0.30 |
| Flock Size | -0.15 | -0.36 | -0.13 |
| Period 1 x Control | reference |  |  |
| Period 2 x Treatment | 0.18 | -0.03 | 0.40 |
| Period 3 x Treatment | -0.18 | -0.41 | 0.05 |
| Period 1 x Flock Size | reference |  |  |
| Period 2 x Flock Size | 0.19 | 0.03 | 0.36 |
| Period 3 x Flock Size | 0.24 | 0.06 | 0.41 |
| Control x Flock Size | reference |  |  |
| Treatment x Flock Size | 0.21 | 0.07 | 0.35 |
| Period 1 x Control x Flock Size | reference |  |  |
| Period 2 x Treatment x Flock Size | -0.03 | -0.25 | 0.18 |
| Period 3 x Treatment x Flock Size | -0.42 | -0.69 | -0.15 |
| Year | 0.03 | -0.27 | 0.32 |
| Error for random species effect | 0.43 | 0.29 | 0.66 |
| Error for random site effect | 0.39 | 0.25 | 0.63 |

Table S2: Coefficient means and 95% Confidence Intervals (CIs) for the model evaluating foraging behavior of feeder birds in Chicago, IL. Estimates for categorical variables and interactions were calculated against the reference listed.

| Parameter | Mean | 2.5% CI | 97.5% CI |
| --- | --- | --- | --- |
| Pre-playback Period | -0.18 | -0.44 | 0.06 |
| Playback Period | -0.39 | -0.79 | 0.37 |
| Post-playback Period | -0.25 | -0.65 | 0.50 |
| Control | reference |  |  |
| Treatment | 0.00 | -0.13 | 0.13 |
| Time of Day | -0.05 | -0.09 | 0.00 |
| Min. Temperature | 0.01 | -0.05 | 0.06 |
| Duration | 0.05 | 0.01 | 0.09 |
| Body Mass | 0.03 | -0.12 | 0.18 |
| Period 1 x Control | reference |  |  |
| Period 2 x Treatment | -0.46 | -0.67 | -0.24 |
| Period 3 x Treatment | -0.17 | -0.37 | 0.03 |
| Period 1 x Body Mass | reference |  |  |
| Period 2 x Body Mass | -0.21 | -0.37 | -0.04 |
| Period 3 x Body Mass | -0.11 | -0.27 | 0.05 |
| Control x Body Mass | reference |  |  |
| Treatment x Body Mass | 0.13 | -0.02 | 0.28 |
| Period 1 x Control x Body Mass | reference |  |  |
| Period 2 x Treatment x Body Mass | -0.20 | -0.46 | 0.05 |
| Period 3 x Treatment x Body Mass | -0.21 | -0.45 | 0.04 |
| Year | -0.14 | -0.37 | 0.10 |
| Error for random species effect | 0.04 | 0.11 | 0.35 |
| Error for random site effect | 0.07 | 0.17 | 0.42 |
